# Circulating Soluble Urokinase-Type Plasminogen Activator Receptor Levels Reflect Renal Function in Newly Diagnosed Patients with Multiple Myeloma Treated with Bortezomib-Based Induction

**DOI:** 10.3390/jcm9103201

**Published:** 2020-10-03

**Authors:** Evangelos Terpos, Ioannis Ntanasis-Stathopoulos, Gerasimos-Petros Papassotiriou, Efstathios Kastritis, Alexandra Margeli, Nikolaos Kanellias, Evangelos Eleutherakis-Papaiakovou, Magdalini Migkou, Despina Fotiou, Maria Roussou, Maria Gavriatopoulou, Panagiotis Malandrakis, Erasmia Psimenou, Ioannis Papassotiriou, Meletios A. Dimopoulos

**Affiliations:** 1 Department of Clinical Therapeutics, School of Medicine, National and Kapodistrian University of Athens, 11528 Athens, Greece; johnntanasis@med.uoa.gr (I.N.-S.); akis.papassotiriou@gmail.com (G.-P.P.); ekastritis@gmail.com (E.K.); nick.kanellias@gmail.com (N.K.); mdeleutherakis2@gmail.com (E.E.-P.); mmigou@gmail.com (M.M.); desfotiou@gmail.com (D.F.); mroussou@med.uoa.gr (M.R.); mariagabria@gmail.com (M.G.); panosmalandrakis@gmail.com (P.M.); erasmia7@otenet.gr (E.P.); mdimop@med.uoa.gr (M.A.D.); 2 Department of Clinical Biochemistry, “Aghia Sophia” Children’s Hospital, 11527 Athens, Greece; margelial@yahoo.com (A.M.); ipapassotiriou@gmail.com (I.P.)

**Keywords:** suPAR, soluble urokinase-type plasminogen activator receptor, multiple myeloma, bortezomib, induction, kidney, renal

## Abstract

(1) Background: Soluble urokinase-type plasminogen activator receptor (suPAR) has been implicated in the pathogenesis of kidney disease in different disease settings. The aim of this study was to investigate a possible link between suPAR circulating levels and renal impairment (RI) in newly diagnosed patients with symptomatic multiple myeloma (NDMM) before and after frontline therapy with bortezomib-based regimens. (2) Methods: We studied 47 NDMM patients (57% males, median age 69.5 years) before the administration of anti-myeloma treatment and at best response to bortezomib-based therapy. suPAR was measured in the serum of all patients and of 24 healthy matched controls, using an immuno-enzymatic assay (ViroGates, Denmark). (3) Results: suPAR levels were elevated in NDMM patients at diagnosis compared to healthy individuals (*p* < 0.001). suPAR levels strongly correlated with disease stage (*p*-ANOVA < 0.001). suPAR levels both at diagnosis and at best response negatively correlated with estimated glomerular filtration rate (eGFR) values (*p* < 0.001). Interestingly, no significance changes in suPAR levels were observed at best response compared to baseline values (*p* = 0.31) among 18 responding patients with baseline eGFR < 50 mL/min/1.73 m^2^. (4) Conclusions: SuPAR levels reflect renal function in NDMM patients treated with bortezomib-based induction. Responders may have elevated circulating suPAR levels, possibly reflecting persistent kidney damage, despite their renal response.

## 1. Introduction

Multiple myeloma (MM) is a multisystemic plasma cell dyscrasia that primarily affects bone homeostasis, hematopoiesis and renal function [1,2,3]. Renal impairment is a common complication of MM and up to 50% of patients with MM present with renal impairment at diagnosis, depending on the definition of renal impairment based on the value of serum creatinine or estimated glomerular filtration rate (eGFR) [4,5]. Prompt initiation of anti-myeloma treatment is of paramount importance in order to restore renal function, whereas the introduction of proteasome inhibitors, such as bortezomib, and other novel agents in the therapeutic algorithm of MM has led to a significant improvement in the survival of patients with severe renal impairment [6,7].

Soluble urokinase-type plasminogen activator receptor (suPAR) is the circulating form of a glycosyl-phosphatidylinositol-anchored three domain membrane protein that has been implicated in the pathogenesis of kidney disease of different etiology [8,9]. Under physiological conditions, low expression of urokinase plasminogen activator receptor is shown on podocytes, endothelial cells and activated immune cells [10]. However, elevated suPAR levels have been consistently associated with decreased renal function in different disease settings including chronic kidney disease, critical illness, sepsis, cardiac surgery and coronary angiography [8,11]. High suPAR levels result in aberrant activation of αvβ3 integrin in kidney podocytes, which ultimately leads to proteinuria and kidney damage [12]. Furthermore, uPAR-mediated signaling has been implicated in inflammation, endothelial damage, as well as in cell migration, adhesion, and mitosis in cancer [13].

In this context, the aim of this study was to investigate a possible link between suPAR levels and renal impairment in newly diagnosed patients with newly diagnosed patients with symptomatic multiple myeloma (NDMM) before and after frontline therapy with bortezomib-based regimens.

## 2. Patients and Methods

suPAR was measured in the serum samples of consecutive patients with NDMM treated with bortezomib-based upfront regimens in a single institution (Department of Clinical Therapeutics, National and Kapodistrian, University of Athens, Greece). Each patient had two measurements: one at baseline before the administration of any kind of therapy, including dexamethasone, and one after best-response to first-line treatment. suPAR levels were also evaluated in apparently healthy individuals of similar age, gender and body mass index, who had donated their blood in the institutional blood bank (controls).

Patient data were collected in a prospectively maintained database and treatment outcomes were assessed according to the International Myeloma Working Group (IMWG) guidelines [14,15].

Measurements of suPAR and other analytes were performed by means of immune-enzymatic techniques as follows: suPAR (ViroGates A/S, Birkerod, Denmark), Neutrophil Gelatinase-Associated Lipocalin (NGAL) (R&D Systems, Minneapolis, MN, USA); whereas Cystatin-C was measured with an immunoturbidimetric assay using the Roche Cobas 6000 Clinical Chemistry System. Apart from markers of renal function (Cystatin-C) and renal injury (NGAL), biomarkers of inflammation (hs-CRP and IL-6) and cardiac function (hs-Troponin-T and NT-proBNP) were also evaluated. eGFR values were calculated based on the Chronic Kidney Disease Epidemiology Collaboration Cystatin-C (CKD-EPI-CysC) equation [16].

The study was conducted according to the principles of the 18th World Medical Association Assembly (Declaration of Helsinki, 1964) and all its future amendments. The study protocol was designed and executed according to the Good Clinical Practice Guidelines as defined by the International Conference of Harmonization (GCP-ICH), as well as the regulations pertaining to clinical studies in Greece. It was approved by the institutional ethics committee.

Statistical analyses were performed with IBM SPSS v.22 statistical software (New York, NY, USA).

## 3. Results

### 3.1. Patient Characteristics

Forty-seven patients with NDMM were included in the study. Twenty-seven (57%) were male and 20 were female (43%), and the median age of the whole study population was 69.5 years. Thirty (64%) patients had a MM diagnosis of IgG isotype, 7 (15%) of IgA and 10 (21%) had light-chain only MM. Regarding the prognostic classification according to the International Staging System (ISS) for MM, 13 (28%) patients had ISS-1, 19 (40%) had ISS-2 and 15 (32%) had ISS-3 MM. All patients received bortezomib-based frontline therapy as follows: bortezomib, cyclophosphamide, dexamethasone (VCD) *n* = 32 (68%); bortezomib, thalidomide, dexamethasone (VTD) *n* = 7 (15%); bortezomib, melphalan, dexamethasone (VMP) *n* = 7 (15%); bortezomib, dexamethasone (VD) *n* = 1 (2%). Twenty-seven (58%) patients had baseline eGFR at diagnosis < 60 mL/min/1.73 m^2^, 23 (49%) had baseline eGFR < 50 mL/min/1.73 m^2^ and 10 (21%) had baseline eGFR < 30 mL/min/1.73 m^2^; whereas no patient was on dialysis.

In the study were also included 24 healthy individuals of similar age, gender and body index, who served as controls.

### 3.2. SuPAR and Other Biomarkers in MM and Controls

**Figure 1:**
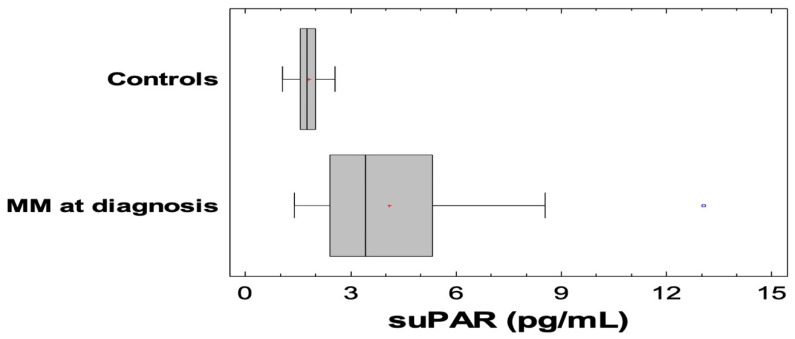
Soluble urokinase-type plasminogen activator receptor (SuPAR) levels were significantly elevated in newly diagnosed patients with symptomatic multiple myeloma (NDMM) patients compared to controls (*p* < 0.001).

suPAR levels were elevated in MM patients at diagnosis compared to 24 healthy individuals [mean±standard deviation (SD) (range): 4.1 ± 2.2 pg/mL (1.4–13.0 pg/mL) versus 1.8 ± 0.3 pg/mL (1.1–2.6 pg/mL), *p* < 0.001] (Figure 1). All other markers of cardio-renal dysfunction (hs-Troponin-T, NT-proBNP, Cystatin-C, NGAL) and inflammation (hs-CRP and IL-6) were elevated in NDMM patients compared to healthy controls (*p* < 0.01 for all comparisons).

### 3.3. Correlations of suPAR with Disease Characteristics, Renal Function during Upfront Treatment, Cardio-Renal and Inflammatory Biomarkers

**Figure 2:**
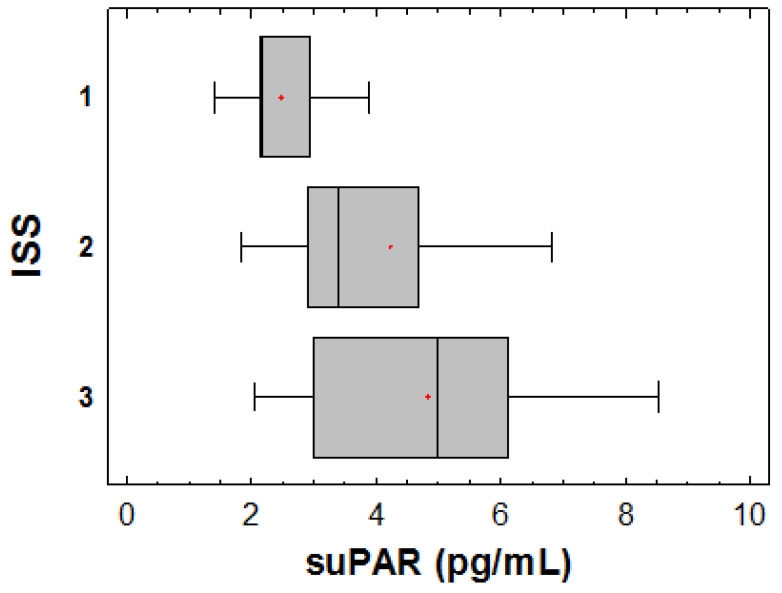
suPAR levels according to MM International Staging System (ISS) stage (Kruskal-Wallis Median Test, *p* = 0.002).

suPAR levels strongly correlated with ISS disease stage (Figure 2). Patients categorized as ISS-1 had a mean ± SD value of 2.4 ± 1.2 pg/mL, whereas the mean ± SD value of suPAR was 3.6 ± 1.8 pg/mL among patients with ISS-2 disease and 5.1 ± 2.2 pg/mL among those with ISS-3 MM (*p*-ANOVA < 0.001). No association was found between suPAR levels and the amount of monoclonal protein.

Following bortezomib-based frontline therapy, 39 patients responded (83%). Nine patients (19%) achieved a complete response (CR), 11 (23%) a very good partial response (VGPR) and 19 (40%) a partial response (PR). Among the 23 NDMM patients with baseline eGFR < 50 mL/min/1.73 m^2^, 18 (78%) showed at least a minor renal response to bortezomib-based upfront regimen. However, no significance changes in suPAR levels were observed at best response (4.4 ± 2.7 pg/mL) compared to baseline values (*p* = 0.31).

**Figure 3:**
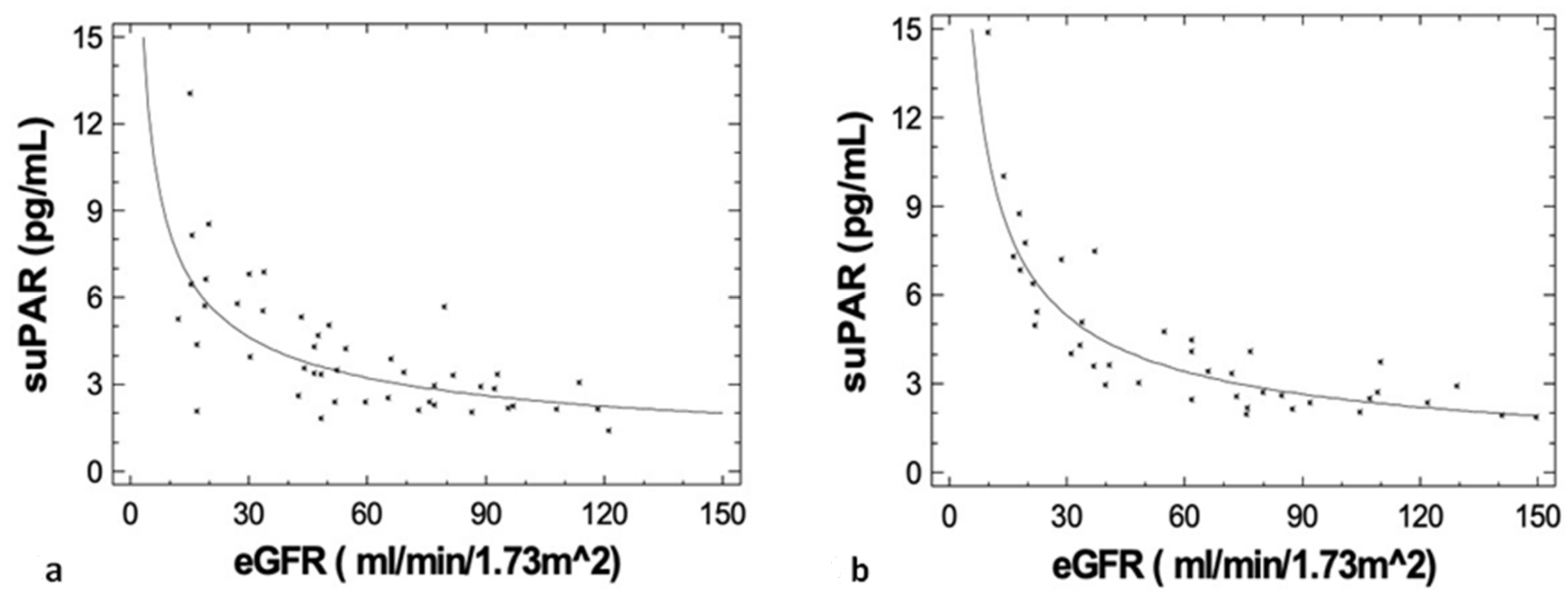
SuPAR levels were negatively associated with estimated glomerular filtration rate (eGFR) values both (**a**) at diagnosis (r = −0.700, *p* < 0.001) and (**b**) at best response (r = −0.890, *p* < 0.001).

**Figure 4:**
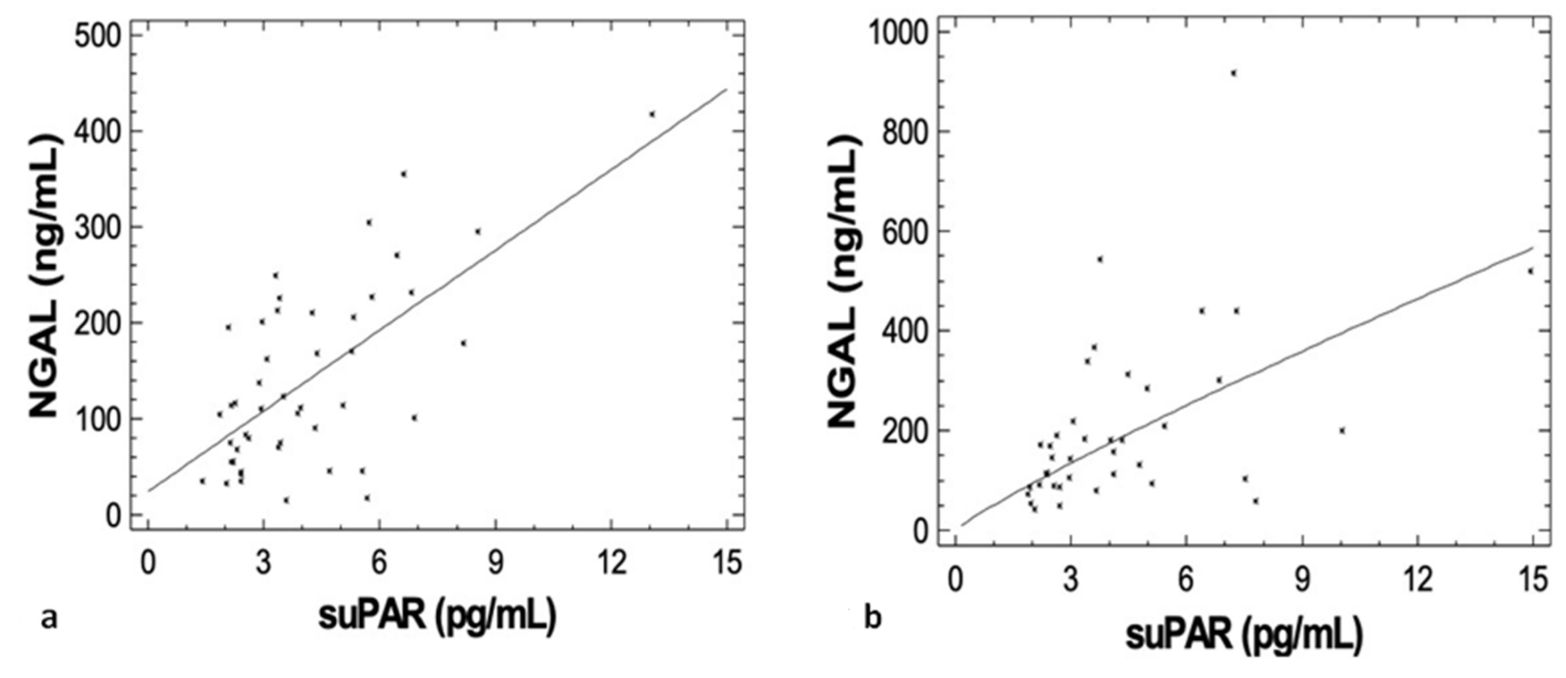
SuPAR levels were positively associated with Neutrophil Gelatinase-Associated Lipocalin (NGAL) values both (**a**) at diagnosis (r = 0.657, *p* < 0.001) and (**b**) at best response (r = 0.586, *p* < 0.001).

Interestingly, suPAR levels strongly correlated with eGFR values both at diagnosis (r = −0.700, *p* < 0.001) and at best response (r = −0.890, *p* < 0.001), (Figure 3a,b, respectively). SuPAR levels were also associated with NGAL values both at diagnosis (r = 0.657, *p* < 0.001) and at best response (r = 0.586, *p* < 0.001) (Figure 4a,b, respectively).

Furthermore, suPAR levels at diagnosis and at best response correlated positively with the (log)values of inflammatory biomarkers IL-6 and hs-CRP (*p* < 0.001 for all correlations), as well as with the markers of cardiac function hs-Troponin-T and NT-proBNP (*p* < 0.001 for all correlations).

## 4. Discussion

Herein, we investigated the association between suPAR levels and disease characteristics in NDMM patients before and following the administration of frontline therapy with bortezomib-based regimens. SuPAR levels were significantly elevated in NDMM patients compared to controls, as well as other markers of cardio-renal dysfunction and inflammation. We also found that suPAR levels were significantly associated with biomarkers of inflammation both at diagnosis and at best response. A disequilibrium favoring the overproduction of inflammatory cytokines results in an inflammatory state in patients with MM, which is reflected as an increase in relevant biomarkers [17]. The expression of uPAR is up-regulated in activated immune cells, which in turn results in increased suPAR levels [18]. This has been reported consistently in studies showing that increased suPAR is associated with systemic inflammation and adverse prognosis in patients with infectious diseases [18,19]. Elevated suPAR levels are implicated in different disease settings associated with inflammation including cardiovascular disease, diabetes, rheumatic disease and cancer, whereas suPAR levels may even be predictable of mortality in the general population [20,21,22]. Furthermore, both myeloma cells and myeloid cells residing in the myeloma compartment of the bone marrow niche express uPAR and may contribute to the increased suPAR levels detected in the serum of patients with MM [23,24,25]. uPAR is thought to sustain the homing of malignant plasma cells in the bone marrow along with other adhesion molecules such as CD56 and CD138 [26]. Interestingly, high uPAR expression has been significantly associated with high expression of CD56, CD138, CD38 and CD45 in the bone marrow of patients with MM, as assessed by flow cytometry [27].

Increased suPAR levels have been also reported in patients with other hematological malignancies such as lymphomas and leukemias [26,28]. Deregulation of the uPA-uPAR signaling cascade has been described in both hematological cancer and solid tumors [13]. Activation of the uPA-uPAR axis leads to the activation of JAK-STAT, RAS-PI3K, RAS-MARK intracellular pathways that regulate gene expression and promote cell proliferation, cell migration, invasion, metastasis and angiogenesis [13]. In the extracellular space, activated uPA mediates the conversion of inactive plasminogen to plasmin, which cleaves and activates growth factors and matrix metalloproteases (MMPs) [13]. Plasmin and MMPs degrade the extracellular matrix, which favors cell motility and migration. Other signaling cascades also promote cell migration, such as the SRC tyrosine kinase—inducible nitric oxide synthase axis (iNOS). Interestingly, pharmacological inhibition of MMP-9 and uPAR has resulted in the suspension of cell migration of glioma cells in preclinical studies [29].

Elevated suPAR levels at diagnosis were correlated with advanced ISS stage and, consequently, with adverse disease prognosis. Our results are in line with other studies in the field, including 46 and 40 patients with MM, respectively, showing that high suPAR levels predicted for extra-medullar myeloma involvement, advanced disease stage and poor survival outcomes [27,30].

High suPAR levels were inversely associated with renal function both at diagnosis and at best response in our study. A similar association between baseline suPAR levels and serum creatinine has also been reported in previous studies among patients with MM [27,30]. More recently, suPAR has been proposed as an emerging biomarker for predicting renal outcomes among patients with monoclonal gammopathy of renal significance (MGRS) [31]. MM-related renal impairment is considered among the most challenging MM complications in terms of restoring renal function and improving survival outcomes [5]. Three distinct clinical scenarios are usually seen in patients with MM: functional renal insufficiency which may rapidly respond to prompt anti-myeloma treatment, acute kidney injury due to MM-related cast nephropathy, or chronic kidney disease, especially when comorbidities are present [32]. Renal biopsy may be necessary for the differential diagnosis among MM- and non-MM related renal pathologies [33]. Renal impairment in patients with MM is mainly attributed to monoclonal involved free light chains, which may cause myeloma cast nephropathy and may also have direct toxic effects on kidneys inducing isolated proximal tubule cell cytotoxicity and/or tubulointerstitial nephritis [3,32]. Elevated circulating suPAR levels derived from myeloma and myeloid immune cells may precipitate kidney damage [12,25]. Endothelial damage, which may be also induced by anti-myeloma treatment, may promote renal impairment [34]. Endothelial injury may be reflected on changes in the levels of suPAR, uPA, uPAR and plasminogen-activator inhibitor type 1 (PAI-1), as well as endothelial extracellular vesicles (EV) [35]. A longitudinal assessment of these markers in patients with MM receiving proteasome inhibitors could elucidate the underlying mechanism of treatment-induced endothelial and renal damage [36].

Novel anti-myeloma agents including immunomodulatory drugs and proteasome inhibitors have significantly improved the therapeutic approach by achieving a rapid reduction in free light chains from treatment initiation, which increases the probability of renal recovery [7,37]. Early renal recovery among patients with MM and renal impairment treated with bortezomib-based regimens has been reported consistently among several prospective and retrospective studies [7]. The importance of rapid intervention in order to reduce excessive free light chains lies in the prevention of progressive and irreversible renal damage, and in particular interstitial fibrosis and tubular atrophy [3,32,38,39]. The sustained high suPAR levels even among responding patients with MM and renal impairment in our study may reflect irreversible kidney damage. It may also be a result of persistent suPAR production especially in patients achieving less than VGPR. Therefore, it seems that an early and deep response is essential to optimize renal response and restore renal function, similar to the importance of a rapid, deep and sustainable hematological response for the organ-specific responses in patients with AL amyloidosis [40].

Circulating suPAR levels were also positively associated with NGAL and cystatin-C levels both at diagnosis and at best response. Both NGAL and cystatin-C are sensitive biomarkers of renal function in patients with MM [41]. Cystatin-C may have also a predictive value for renal response to bortezomib-based regimens both among previously untreated and relapsed/refractory patients with MM [42,43]. Furthermore, the association between suPAR levels and markers of cardiac function both at diagnosis and at best response may be at least partially attributed to confounding factors including inflammatory state and renal dysfunction [44].

Compared with previous studies in the field, the serial assessment of circulating suPAR levels both before and post bortezomib administration in consecutive patients with NDMM is among the novelties of our study. Furthermore, the assessment of renal function based on the CKD-EPI-CysC equation, instead of Modification of Diet in Renal Disease (MDRD), Cockroft-Gault or serum creatinine levels as in other studies, adds further value to our results. CKD-EPI-CysC is more sensitive in detecting renal dysfunction among patients with MM compared with MDRD and has also a significant prognostic role [45]. Among the limitations of our study is the relative small number of included patients, especially in the subgroup analyses, as well as the fact that no patient was in need of renal dialysis at presentation and, therefore, we could not evaluate the role of suPAR levels in this patient group. A longitudinal, serial analysis of suPAR levels during the disease course could be of value in order to correlate changes in suPAR levels with treatment response.

In conclusion, circulating suPAR levels are associated with renal function in patients with NDMM both at diagnosis and at best response to bortezomib-based frontline therapy. Importantly, responders to anti-myeloma therapy continued to have elevated circulating suPAR, possibly reflecting underlying permanent kidney damage or persistent production from residual myeloma clones and associated myeloid immune cells. SuPAR has emerged as a useful biomarker of renal function in MM, whereas larger clinical studies may further determine the potential value of its integration in the clinical practice.
